# Recent Advances in the Antiproliferative and Proapoptotic Activity of Various Plant Extracts and Constituents against Murine Malignant Melanoma

**DOI:** 10.3390/molecules27082585

**Published:** 2022-04-17

**Authors:** Daria-Antonia Dumitraș, Sanda Andrei

**Affiliations:** Department of Biochemistry, University of Agricultural Sciences and Veterinary Medicine, Mănăştur no. 3–5, 400372 Cluj-Napoca, Romania; antonia.dumitras@usamvcluj.ro

**Keywords:** malignant melanoma, bioactive compounds, phytotherapy, in vitro, in vivo

## Abstract

Although conventional medicine, chemical drug synthesis and pharmaceutical research are advancing at a rapid pace, nature remains a major supplier of biological molecules. Natural bioactive compounds are studied closely especially as an alternative to the limitations of conventional therapy in many diseases, melanoma being one of them. Malignant melanoma is a highly aggressive type of cancer, and the current methods of treatment used are cryotherapy, external surgery, radiation therapy, chemotherapy, photodynamic therapy, biological therapy, and targeted drug therapy. Unfortunately, these treatment methods are often inefficient, extremely expensive and cause many side effects, which is why focusing on melanoma chemoprevention and adjuvant therapy with natural herbal phytoconstituents is an emerging strategy to prevent, cure or treat melanoma. This review aims to examine the latest discoveries in terms of potential natural bioactive compounds that possess important activity against the development and spread of murine melanoma cancer. In particular, the use of different phytochemicals such as phenolic acids, flavonoids, anthocyanins, terpenoids, essential oils and carotenoids in vitro and in vivo models will be discussed. These data are helpful in guiding researchers in the direction of studying phytonutrients with important effects in the prevention and treatment of melanoma.

## 1. Introduction

Plants are considered to be the richest source of novel chemical compounds; hence they became the main attraction for researchers around the world wanting to discover new chemotherapeutic candidates derived from natural products. At the moment, over 75% of the currently used antineoplastic drugs are derived from natural sources including marine, microbial, and botanical sources [1,2,3]. Historically, natural products played a powerful role in the treatment of both animal and human illnesses. At present, natural products include a large part of current pharmaceutical agents, mostly in the field of cancer therapy [4].

### 1.1. Incidence and Epidemiology

Clinical data show that skin cancer occurs mostly based on geographical areas. Basal cell carcinoma (BCC) is usually reported among Hispanics, Caucasians, Japanese, and Chinese Asians, while squamous cell carcinoma (SCC) is more common among African Americans, Americans, and Asian Indians. In contrast, melanoma is less common, but is life-threatening. Malignant melanoma is the 19th most commonly reported skin neoplasia. In the past decade (2011–2021), the amount of new invasive melanoma diagnosed annually increased by 44% according to Skin Cancer Foundation. According to the American Cancer Society, skin cancer is by far the most common of all cancers, and although melanoma accounts for only about 1% of skin cancers, it leads to a large number of deaths. The rates of melanoma have been rising rapidly over the past few decades, but this has varied by age. Melanoma has been reported to be 20 times more common in white people than in black people, and the average age of diagnosis is 65 years old. Before the age of 50, more women are diagnosed with melanoma than men [5].

The American Cancer Society’s approximations for melanoma in the United States for 2022 are: about 99,780 new melanomas will be diagnosed (about 57,180 in men and 42,600 in women), and about 7650 people are expected to die of melanoma (about 5080 men and 2570 women) [6].

Not only humans are affected by this severe type of cancer, but also small animals, mainly dogs. A survey over a period of 6 years on neoplasms in small animals diagnosed 2154 neoplasms, out of which 8.9% were melanomas (*n* = 193). Canine species were the most affected, accounting for 96.4% (*n* = 186), whereas cats represented only 3.6% (*n* = 7) of the total number of melanomas diagnosed [7].

It is well known that malignant melanoma is a highly aggressive malignant melanocytic neoplasm with increasing rates every year and an etiology that derives from the transformation and uncontrolled growth of melanocytes, and which is resistant against most conventional therapies, resulting in a growing public health problem [8]. Malignant melanoma is the result of epidermal melanocytes and is a metastasis-prone malignancy [9]. Melanin is a pigment that is biosynthesized from L-tyrosine in melanocytes and plays an important role in preventing skin cancer caused by ultraviolet rays; hence the need of focusing on finding natural melanogenesis modulators. Numerous risk factors for the expansion of melanoma have been acknowledged, including family history of melanoma, genetic susceptibility, environmental factors, ultraviolet radiation, and age-related immune suppressions. Melanomas include many genotypic and phenotypic subtypes that can promote and better adapt to survive in extreme environmental conditions, and are among the tumors with greatest genomic instability. Moreover, melanomas that infiltrate the dermis can produce metastases, and are most often responsible for the high mortality rate of the ailment [10]. Taking into consideration the complexity of the tumor cells, cutaneous melanomas are often challenging when it comes to treatment, despite the progress that has been made toward the combined application of surgical operation and chemotherapies. Therefore, developing a novel treatment or strategy to combat the disease has become an urgent matter [11].

### 1.2. Current Treatment Options in Malignant Melanoma

Current methods of treatment for melanoma cancers include cryotherapy, external surgery, radiation therapy, chemotherapy, photodynamic therapy, biological therapy, and targeted drug therapy such as vemurafenib, dabrafenib, and trametinib [12]. The development of multidrug resistance, multiple side effects, and the high costs of the therapy existent on the market at the moment for malignant melanoma are important grounds for the need to discover new compounds that are safe and more effective, recognizing that the current solutions used to be unsatisfactory due to the pitfalls of these treatment methods.

In conclusion, focusing on the study of natural herbal phytoconstituents represents an emerging strategy in order to help in the prevention and treatment of melanoma in the near future. A colossal advantage of plant-derived compounds is represented by the multitude of their effects, possessing active compounds that affect cell proliferation, apoptosis, metastasis, and angiogenesis, and which can be used against cancer securely, efficiently, and with negligible side effects [13,14,15,16]. Additionally, their protective capacity against some side effects of radio and chemotherapy is not to be neglected [17,18].

To screen antitumor agents, multiple cell lines have been created with the purpose of producing animal models of cancer. Regarding melanoma, there are multiple types of cell lines, among which are melanoma B16F10 (metastatic) and B16F0 (non-metastatic), both of which originate from C57Bl/6 mice; B16F0 represents the parent cell line. The B16F10 model is a very aggressive one at the biological level, and is most often used in research [19].

The main aim of this review is to provide a comprehensive summary of the most recently discovered natural products that possess important activity against murine malignant melanoma. This information is valuable and represents the first step in the research of effective phytonutrients against the development and spread of murine melanoma, because based on this, further research can be performed in order to discover bioactive compounds effective in the prevention and treatment of melanoma.

## 2. Plant-Derived Drugs: A Historical Perspective

Plants have long served humankind as a source of medicinal substances. In fact, natural products once served as the source of all medicine. Even today, natural products (and their derivatives and analogs) make up more than 50% of all clinically used drugs. Ayurveda, Chinese medicine (TIM), and Chinese medicine (TCM) are the oldest (4500 BC) still-living traditions. In ancient times, knowledge about how to choose the right plants, when to collect them, and how to prepare medicines for specific purposes was verbally passed from generation to generation. The last century has seen significant advances in the field of plant and microbiology research, with the expansion of several compounds employed in cancer treatment protocols. Many successful anti-cancer drugs that have been used clinically and have shown significant effects are derived from natural products such as plants, marine organisms, and microorganisms. Recent advances in proteomics and metabolomics have helped identify new therapeutic targets that open up new therapeutic prospects. Over 50% of all molecules approved between 1981 and 2014 are natural products or derived from them, and most serve as antitumor agents [20]. In this context, natural products have been recognized as a rich and successful source for the development of new cancer therapies [21].

The present review emphasizes the importance of the discovery of new plant-derived metabolites for use in cancer therapeutics and highlights the anticarcinogenic potential of natural products.

## 3. Sources and Methodology

All the data were achieved through a broad search of electronic databases including Science Direct, Google, Google Scholar, PubMed, SCOPUS, and Web of Science Core Collection. The search was restricted to the English language only. The search terms or keywords used were B16F10 melanoma, cancer phytotherapy, plant extracts, antitumoral natural substances, botanical extracts with anticancer properties, natural products against cancer, natural substances, and B16F10 melanoma, and natural anticancer molecules. In almost all cases, the original articles were obtained, and the relevant data were extracted. In the first step of the review, the titles and abstracts of the returned articles were analyzed. The potentially eligible articles had to be published in the last 10 years, between the 1st of January 2012 and the 31 of December 2021, and as expected, an impressive number of studies focused on our topic of interest were published in that time frame.

In the second step of the review, the full text of the potentially eligible articles, which had to be available for reading or purchasing, was analyzed. Detailed information about the studied plants, their biochemical characterization, and any tests performed in vitro and/or in vivo focusing on the antiproliferative effect of the natural substances on B16F10 melanoma cells were reported. All the final articles had to have the references listed and had to have been cited at least once, except for very recently published ones (during the last 6 months). The number of citations was determined using the Web of Science Core Collection search tool and, where the articles were not available, the Google Scholar search engine was employed.

Table 1 presents a comprehensive list with information about each of the plants contained in the eligible articles, their main bioactive components, and their mechanism of action.

## 4. Bioactive Compounds Tested on B16F10 Murine Melanoma Cells—In Vitro

The research conducted over the last decade has discovered a large plethora of pharmacological activities induced by phytotherapeutic agents.

### 4.1. Polyphenols

Polyphenols, one of the most important phytochemicals ubiquitously found in herb plants, vegetables, and fruits, represent a very important area for the entire scientific community, with a vast volume of literature being focused on the research of these compounds, mostly due to the important contribution they are making to finding new treatments for one of the most important diseases [77,78,79,80,81]. Phenolic compounds possess remarkable properties, with anti-inflammatory, antimicrobial, antiviral, anticancer, and immunomodulatory properties having been reported [80]. Due to their potential in modulating various mechanisms related to carcinogenesis, polyphenols can be considered to be strong cancer inhibitors and preventives. Multiple studies in vitro and in vivo have demonstrated their antioxidant activity, cell survival pathway regulation associated with cell proliferation, differentiation, migration, angiogenesis, detoxification enzymes, and immune responses [77,82].

Notwithstanding their vast capabilities in cancer prevention and treatment, polyphenols have the disadvantage of poor bioavailability, and poor absorption and biodistribution, as well as fast metabolism and excretion [77]. A solution found by scientists to counteract these shortcomings was a combination of more polyphenols or polyphenols and other anti-neoplastic drugs, thus avoiding multiple side-effects that conventional drugs might cause [80]. Consequently, polyphenols showed their chemopreventive ability against squamous cell carcinoma and lung cancer in multiple studies [78,79].

Polyphenols have been reported to be the main bioactive compounds by researchers investigating multiple extracts from *Cynomorium coccineum* L. (Maltese Mushroom) in 2016 as follows: water extract from the whole plant (WP), external layer (EL), and peeled plant (PP) containing polyphenols in doses between 25 and 500 μg/mL. As a result, the authors described reduction in cell viability by 41% and 68% when the cells were treated with 250 and 500 μg/mL WP extract solutions, respectively. EL extract determined a reduction in viability up to 49%. Although the PP extract seemed to be less effective than the EL and WP extracts, the cell viability went down by 46% at a concentration of 500 μg/mL [23].

As Chao and collaborators (2022) concluded in their study [24], when concentrations of polyphenols between 100 to 200 μg/mL were used to treat B16F10 cells, obvious inhibitory effects were noticed. Additionally, the intracellular activity of tyrosinase decreased significantly by up to 88.63%, 85.57% and 81.44%, respectively, after incubation with 200, 600 and 800 μg/mL of hull-less pumpkin polyphenols. In addition, hull-less pumpkin (*Cucurbita maxima* Duch.) polyphenols reduced cellular melanin production under the stimulation of α-MSH. The inhibition rate of melanin production decreased to 88.23%, 83.56% and 76.61% at when incubated with 200, 600 and 800 μg/mL of polyphenols. Another valuable result was published by Pin-Hui Li and collaborators in 2016, who tested five types of extracts as follows: ethyl acetate (EA), dichloromethane (DM), n-hexane (Hex), methanol (MeOH) and water extract. Although none of the extracts had a promising effect on B16F10 cell proliferation according to the MTT assay performed, EA extract inhibited cellular melanin production and DM extract enhanced cellular pigmentation. EA extract decreased melanin production by 32% at 200 μg/mL. In contrast, 200 μg/mL DM extract substantially amplified melanin production (by 112%), and the effects of DM extract were dose dependent. The Western blot analyses performed showed that EA and DM extracts affected melanin content by regulating MITF (microphthalmia-associated transcription factor), tyrosinase, TRP-,1 and TRP-2. EA extract concentrations ranging from 10 to 100 μg/mL reduced expressions of MITF, tyrosinase, TRP-1, and TRP-2 in a dose-dependent manner. Moreover, DM extract promoted expressions in B16-F10 cells in a dose-dependent manner.

*Moringa oleifera* Lam. (MN), *Eremomastax speciosa* (Hochst.) Cufod (ES) and *Aframomum melegueta* K. Schum (AM) extracts were individually analyzed to determine their possible effect on murine melanoma [25]. Following treatments for 48 h with each of the three extracts, inhibition of cell proliferation was observed, of 16%, 32% and 39%, respectively. After 72 h, the inhibition percentage increased to 42% (MN), 47% (ES) and 61% (AM). Moreover, an increase in the G2/M phase (10.9%) and also cell cycle in the G1 phase (13.9%) were described. Levels of p53 were enhanced by MN, ES, and AM extracts with 50.8%, 28.8% and 40.1%, in that order. The authors considered this to consequently stimulate p21^WAF1/Cip1^- and p27^Kip1^-dependent cell cycle arrest, with a notable increase in p27^Kip1^ in the MN sample. All of these effects lead to different percentages of apoptosis in B16F10 cells. More recently [27], *Caesalpinia spinosa* plant (P2Et), which is abundant in phenolic compounds such as gallic acid (GA) and ethyl gallate (EG), was studied. Showing a higher cytotoxic activity, EG was reported with an inhibitory concentration (IC50) of 23.6 µg/mL (119.1 μM), while GA had an IC50 of 83.5 µg/mL (490.83 µM). Treatment with both compounds led to cellular apoptosis accompanied by autophagy in B16F10 cells, as demonstrated by a rise in the number of LC3 puncta, which was associated with heightened creation of autophagic vacuoles.

Huang and collaborators (2020) evaluated the way melanogenesis was affected after treatment of melanoma cells with *Phoenix dactylifera* L. seed phenolic extract [30]. As a result, intracellular ROS (reactive oxygen species) content decreased; also, the expressions of melanocortin 1 receptor (MC1R), MITF, tyrosinase, tyrosinase-related protein-1 (TRP1), and tyrosinase-related protein-2 (TRP2) were significantly decreased and melanogenesis was inhibited, although no cytotoxic effect was noticed upon the treatment of B16F10 melanoma cells. The researchers concluded that the mechanism by which P. dactylifera exerted these effects on the murine melanoma cells was by downregulating protein kinase A (PKA) signaling pathways.

Data regarding the antioxidant and anti-proliferative potential of *Remirea maritima* hydroalcoholic (RMHA) extracts after testing on murine melanoma cells was reported for the first time in 2015 [32]. The authors correlated these effects with the high total phenolic content with redox properties contained in the tested plant.

Experiments conducted by a group of researchers from Brazil [34] revealed the high anticancer potential of a phenolic extract from *Viscum album* tinctures. Apoptosis induction, cell cycle effect, and antiproliferative effect were described in their report, corroborating increased Sub G0 population, most likely associated with an important reduction in G1, and an increase in S or G2/M populations. Recently discovered as an anti-melanoma agent [36], ethanolic extracts obtained from *Syringa vulgaris* L., especially those from flowers and leaves, exhibit a significant cytotoxic activity, most probably due to the high content of active components belonging to various curative important structural classes (phenil-propanoids, flavonoids, secoiridoids). Following treatment, a significant decrease in viable cell number was observed, and a high antioxidant capacity was demonstrated.

To find new natural therapeutic agents that can prevent and treat malignant melanoma, a multitude of studies regarding the effects of phytonutrients on this type of cancer-focused on plants containing an important amount of polyphenols, both phenolic acids, and flavonoids.

A massive study was conducted to evaluate the effect of 582 methanolic extracts obtained from a variety of parts of 370 plants [4]. The extracts of the leaves of *Annona squamosa* (Annonaceae), the aerial parts and roots of *Tylophora tanakae* (Asclepiadaceae), and the leaves of *Thuja occidentalis* (Cupressaceae) showed the most potent anti-proliferative activities against all cell lines. Anti-proliferative activities of the plants against B1610, the most potent ones listed were: fruit extracts from *Actinidia chinensis* (Actinidiaceae family), bulbs extract from *Allium sativum* var. *pekinense* (Amaryllidaceae family), bark, leaves and seeds extract from *Mangifera indica* (Anacardiaceae family), leaves extract from *Annona cherimola* (Annonaceae family), leaves and stems extracts from *Annona muricata* (Annonaceae family), fruit, leaves and roots extracts from *Anthriscus sylvestris* (Apiaceae family), aerial parts extracts from *Osmorhiza aristate* (Apiaceae family), fruits extracts from *Torilis japonica* (Apiaceae family), barks extract from *Aralia elata* (Araliaceae family), leaves extract from *Araucaria heterophylla* (Araucariaceae family), leaves and twigs extract from *Tylophora ovata*, leaves extract of *Tylophora ovata* var. *brownie*, aerial parts and roots extract from *Tylophora tanakae* (Asclepiadaceae family), leaves extract from *Dracaena draco* (Asparagaceae family), roots extract from *Crepidiastrum lanceolatum*, roots extract from *Saussurea lappa* (Asteraceae family), roots, stems and leaves extracts from *Berberis japonica* (Berberidaceae family), barks extract from *Garcinia subelliptica* (Clusiaceae family), seeds extract from *Luffa acutangular* and *Momordica cochinchinensis* (Cucurbitaceae family), leaves extract from *Juniperus rigida* and *Thuja occidentalis* (Cupressaceae family), leaves extracts from *Persea americana* (Lauraceae family), rhizomes extracts from *Coptis japonica* (Ranunculaceae family) and leaves, stems, and twigs extracts from *Cephalotaxus harringtonia* (Taxaceae family). The same authors isolated six polyphenols from the seeds of *Rhynchosia volubilis* (Fabaceae) that showed a strong inhibition effect on B16F10 melanoma cells. Eleven (11) flavones isolated from the leaves of *Lantana montevidensis* (Verbenaceae), five active sesquiterpenes from the roots of *Inula helenium* (Asteraceae), ten triterpenes that were isolated from the aerial parts of *Centella asiatica* (Apiaceae), possessed a similar effect. In addition, an acridone alkaloid (1,3-Dihydroxy-4-(2′-hydroxy-3′- hydroxymethyl-3′,4′-epoxybutyl)- N-methylacridone) showed very strong anti-proliferative activity.

### 4.2. Flavonoids

Flavonoids are broadly distributed in plant flora, with over 8000 varieties of flavonoids described, divided into six main groups: flavones, flavanones, flavonols, flavanols, isoflavonoids, chalcones, and anthocyanidins [83,84]. Due to their antioxidant, anti-inflammatory and anti-carcinogenic properties, flavonoids are already part of many pharmaceutical, medical, and cosmetic applications. Their antiproliferative and pro-apoptotic activity both in vitro and in vivo has focused on a wide range of cancer types including breast, colorectal, prostate, kidney, lung, stomach, thyroid, oral and laryngeal, leukemia, melanoma, mechanisms such as carcinogen inactivation, antiproliferation, cell cycle arrest, induction of apoptosis and differentiation, inhibition of angiogenesis, anti-oxidation and reversal of multidrug resistance, or a combination of any two or more of these mechanisms underlying anticancer effects [8,85].

In a recent study [28], the effect of *Anastatica hierchuntica* extract (AHEs) on B16F10 melanoma was investigated, concluding that AHE could represent an active ingredient in future treatment protocols for melanoma. The high concentration of phenolic acids and flavonoids in the extracts proved to have a dose-dependent inhibitory effect on the viability of melanoma cells, with the use of concentrations between 400 and 500 μg/mL being the most efficient. Significant inhibition of ROS generation was also observed. Apoptosis and genotoxicity of B16F10 were confirmed by the morphological features of the treated cells. *Abeliophyllum distichum Nakai,* also called white forsythia, is a widely used ornamental plant that has recently been studied more intensely due to its bioactivities, including antioxidant and anti-inflammatory effects. Additionally, DNA damage inhibition, whitening property, anti-diabetic effect, antihypertensive activity, and anti-proliferative activity against human colorectal cancer cells are properties assigned to this plant. The 18 phenolic compounds and the multiple active ingredients, including acteoside, eutigoside B, isoacteoside, rutin, cornoside, hirsutrin, chlorogenic acid, caffeic acid, gentisic acid, ferulic acid, and quercetin, identified in *A. distichum* organ extracts, especially from the leaves (AL), were proved to have a cytotoxic effect at concentrations of 50–200 µg/mL [29]. Nanni and collaborators (2020) investigated the antitumor effect of *Origanum vulgare* L. ssp. *hirtum* hydroalcoholic extract, a Mediterranean plant, emphasizing the molecular bases underlining the antineoplastic effect. Both apoptosis and necrosis, along with oxidative stress, melanogenesis, and tumor cell proliferation inhibition, have been reported by the scientists as phenomena determined by oregano extract treatment [31]. A significant decrease in B16F10 cell growth was noticed after using higher doses of HCOE (hydroalcoholic oregano extract), especially after 48 h of exposure to 6, 8, and 10 mg/mL, respectively. They concluded that oregano extract represents a central candidate as an anticancer agent due to it being highly selective and effective against melanoma cells. The information reported in 2018 [33] provided important knowledge regarding the exhibited properties of *Spartium junceum* L. hydroalcoholic flower extract, most probably thanks to its rich biochemical profile and pro-oxidant activity. According to the authors, *S. junceum* provides melanogenesis inhibition by exerting a pro-oxidant activity and inducing senescence by determining cell cycle arrest in the G2/M phase in B16F10 murine melanoma cells, hence preventing hyper-proliferating events. After 48 h of treatment, an important reduction of B16F10 cell quantity was noticed, especially at high concentrations. Furthermore, the economic advantage this phytocomplex would bring by being an easily available anti-neoplastic drug was underlined.

According to Bouzaiene and collaborators (2016), apigenin-7-glucoside, genkwanin, and naringenin exhibit an important anti-proliferative activity against B16F10 melanoma cells, by increasing sub G0/G1, S and G2/M phase cell proportion with a significant decrease in cell proportion in G0/G1 phases. Furthermore, the authors report apigenin-7-glucoside and naringenin as possessing the ability to enhance the melanogenesis synthesis and tyrosinase activity of B16F10 melanoma cells, whereas genkwanin, by inhibiting tyrosinase activity, can induce a decrease in melanin synthesis [86]. Apigenin has also been reported by other scientists [35], together with sinensetin, as a tyrosinase inhibitor indicating the strong antimelanogenic effect of Pfc (*Perilla frutescens* var. crispa) extract after testing on B16F10 cells. Dana and collaborators showed [34] the strong cytotoxic effect of *Punica granatum* peel extract, from its lowest value (1%) to its highest (61%) in a dose-dependent manner, after previously demonstrating that black pomegranate extract can inhibit angiogenesis in vitro [87]. The calculated IC50 value for the PPE against B16F10 cells was found to be 310.21, indicating that the PEE was toxic against B16F10 cells. The high concentrations of phenolic compounds, flavonoids and tannins were considered to be responsible for these effects. *Lindera obtusiloba* Blume extracts, with the main components quercitrin and afzelin, possess inhibitory effects on tyrosinase activity and melanin synthesis in a concentration-dependent manner [37]. In another study, [8], biflavonoid amentoflavone was described as a compound that possesses pro-apoptotic effect in murine melanoma cells, an effect related to a mechanism with the G0/G1 to S phase arrest, p21, p27, p53, bcl-2 Bax and caspase-3 and -9 protein regulation.

### 4.3. Anthocyanins

Anthocyanins are non-toxic natural pigments and the secondary water-soluble metabolites found in most species in the plant domain, especially flowers, fruits, and tubers. The valuable health effects that they exert and the possible commercial applications they have in the nutraceutical, pharmaceutical, medical and cosmetic industries has to led to an enhanced interest in studying them and their derivates [88,89].

A massive number of types of cancer (colorectal cancer, liver cancer, esophageal cancer, pancreatic cancer, oral cancer, breast cancer, ovarian cancer, thyroid cancer, prostate cancer, bladder cancer, non-small-cell lung cancer, leukemia and melanoma) have been reported to be influenced by anthocyanins, which can exert therapeutic and preventive effects by exerting cytotoxic effects and inducing DNA damage to cause cell cycle arrest. Without doubt, in vivo animal studies or human clinical studies are more convincing in this area [19,90].

Anthocyanin-enriched extract (AEE) obtained from *Sambucus nigra* (elderberry) was characterized by Rugină and collaborators in 2017 [39], with the purpose of establishing a possible anti-proliferative and anti-metastatic effect on B16F10 cells. Strong evidence was found by the authors, who concluded that AEE significantly inhibited proliferation in a concentration-dependent manner, with an IC_50_ of 264.3 µg/mL. Additionally, LDH (lactate dehydrogenase), as a marker of membrane integrity, increased 74% in B16F10 cells treated with 250 µg/mL AEE compared to control. Apoptosis was the mechanism of melanoma cell death. The encouraging results suggested the possibility of elderberry-derived anthocyanins to be utilized in future applications regarding skin cancer. Anthocyanidin and anthocyanin extracted from low-bush wild blueberries (*Vaccinium uliginosum* L.) have also been reported [40] to block cell cycle progression and induce apoptosis in melanoma cells. An important number of anthocyanins have been reported over the time in scientific reports to have the ability of interact with murine melanoma development. Hence, mulberry anthocyanins showed an inhibitory effect on B16F10 melanoma in C57BL/6 mouse model by reducing the MMP-2 and MMP-P expressions [8]. Moreover, malvidin-3-*O*-galactoside, petunidin-3-*O*-galactoside and delphinidin-3-*O*-galactoside, the major anthocyanins from a total of 12, identified [41] from blueberry extracts from different cultivars, showed pro-apoptotic and antiproliferative effects at concentrations higher than 500 µg/mL. Additionally, in 2015, a study regarding the effects of anthocyanin-rich fractions extracted from blueberry and blackcurrant was performed, with important results proving antiproliferative activity [42].

### 4.4. Alkaloids

Alkaloids are nitrogen-containing heterocyclic compounds that possess a strong biological activity. Fundamentally, they are synthesized by plants, amphibians, and marine invertebrates as shields against predators; consequently, most alkaloids are toxic. In recent decades, an extensive range of bioactivities, including antineoplasic, antimicrobial, anti-inflammatory, antinociceptive, has been attributed to these natural compounds [91]. Vinca alkaloids (vinblastine, vincristine, vindesine, vinorelbine) and taxanes (paclitaxel, docetaxel) were the first to be employed clinically in the management of melanoma. However, their downsides are an overall low response rate when used as monotherapy, high toxicity, and the non-negligible resistance of melanoma cells to these compounds [8].

Fofaria and collaborators determined in 2014 [43] the cytotoxic effects of piperine, a major constituent of black and long pepper in melanoma cells. The growth inhibitory effects of piperine were mediated by the cell cycle arrest G1 phase correlated with the down-regulation of cyclin D1 and induction of p21. Additionally, they presented piperine treatment as a generator of ROS in melanoma cells.

### 4.5. Polysaccharides

Naturally occurring polysaccharides are of great interest in the medical field due to their plentiful therapeutic properties, including antitumor and immunomodulatory activity, with relatively reduced side effects, and proven effects both in laboratory studies and in the clinic [8,92]. Researchers have reported, in a study on polysaccharides extracted from *Inonotus obliquus* [45], the tumor growth and invasion suppressing effect possessed by this compound. Chaga mushroom extract decreased expression levels and activities of matrix metalloproteinase (MMP)-2 and MMP-9 by down-regulating the NF-κB signaling pathway, hence preventing the migration and invasion of B16F10. In addition, a reduction in the phosphorylation levels of some kinases (ERK, JNK, MAPK) has also been reported. Additionally, in a recent study conducted in 2021 [44], the biological activity of apple pectin in vitro on two tumoral cell lines was reported, including B16F10 murine melanoma. The enzymatically isolated pectin potently inhibits proliferation, adhesion, and invasion of murine melanoma B16F10 cells, while remaining neutral to non-transformed cells.

### 4.6. Terpenes

Terpenes, also known as terpenoids, are one of the largest and most diverse groups of naturally occurring compounds. They are classified as mono, di, tri, tetra and sesquiterpenes, and are mostly found in plants. Usually, they account for a major constituent of essential oils originating from plants. Among the natural products that provide medical benefits for an organism, terpenes play a major and a variety of roles, anticancer and antidiabetic roles being among them. They are most commonly found in plants like tea, thyme, cannabis, Spanish sage, and citrus fruits (oranges, lemons, limes, mandarins) [93].

Brassicolene, a diterpenoid isolated in red macroalgae (*Gelidium latifolium*) ethanol extract (GLE), showed moderate toxicity and determined changes in the morphology of B16F10 cells such as shrinkages and rounding [47]. A high flavonoid and phenolic acid concentration has also been reported in GLE. In addition, high doses of GLE (100–200 µg/mL) resulted in an increased number of dead cells. Condensed nuclei, nuclear DNA condensation, and chromatin condensation was noticed, with these being one of the key features of cell apoptosis. Apoptosis-related genes were also altered following the explosion to GLE for 72 h. Following treatment with GLE at concentrations above 100 µg/mL, a significant increase in the expression of Bax and Bak was reported, while the expression of Bcl2 was suppressed. This points to the mitochondrial apoptotic pathway as the mechanism of apoptosis.

Maslinic acid (MA), a natural triterpene from *Olea europaea* L., showed significant inhibition of the B16F10 cell viability, up to 20% at the highest dose used (212 µM). Additionally, it prevented oxidative stress caused by H_2_O_2_ excess by decreasing ROS production [53]. Mustapha reported in his study (2015) the anti-proliferative (decreased proliferation by 92.43% after 48 h of incubation) and anti-melanogenic activity ursolic acid (triterpenoid) and vitexin-2′′-*O*-rhamnoside (flavonoid) exerted on B16F10 murine melanoma cells [53]. Furthermore, a decreased tyrosinase activity and a reduced amount of intracellular melanin (1.9 mg per 106 cells and 1.73 mg per 106 cells at doses of 12.5 mg/mL and 5 mM, respectively) when compared with untreated cells (5.6 mg per 106 cells) was reported.

Sea cucumber saponin also displays inhibitory effects on melanoma cells when co-administrated with dacarbazine, diminishing the viable cells by up to 50% [49].

*Zanthoxylum rhetsa,* a medium-sized aromatic tree that has been used by the Kannikar tribes from Tamil Nadu to treat pain, but which is also known for its antimicrobial, antiseptic and astringent properties, was studied recently [12]. The authors analyzed and isolated two tetrahydrofuran lignans (yangambin and kobusin), a berberine alkaloid (columbamine) and a triterpenoid (lupeol) from the bark of this plant. Their results indicated a high percentage of cell death in B16F10 cells, with IC_50_ values ranging from 112.2 (kobusin) to 442.4 µg/mL (yangambin). In addition, both compounds were found to be non-toxic to human dermal fibroblast cells. Lupeol has previously been reported to have the capacity to mediate anticancer activity against melanoma cells by altering the level of Bcl-2, Bax protein, and Wnt/β-catenin signaling. Scientists concluded that the presence of compounds such as lignans and alkaloids plays an important role in the overall cytotoxic effects of *Z. rhetsa* bark extract towards the melanoma B16-F10 cell line [94]. An ethanolic extract from leaves of *Callicarpa longissimi* has been reported to be an inhibitor of melanin production by suppressing microphthalmia-associated transcription factor (MITF) gene expression. Following the treatment of B16F10 cells with the *C. longissima* extract, Western blots revealed a decrease in the levels of MITF and TYR, with carnosol and carnosic acid being considered to be responsible for the major inhibitory effect on melanin production in B16F10 cells [46].

### 4.7. Essential Oils

Essential oils are hydrophobic liquids containing volatile compounds, consisting primarily of terpenes, originating from plants. A multitude of studies determining their antifungal, antibacterial, antibiotic and antiviral properties have been performed, many of them underlining the impressive role essential oils have in modulating tumor growth, and their cancer cell targeting activity [8]. The main role of EOs is to defend against multiple predators; as a result, each plant produces its own specific “signature” mix of EO chemical compounds. It can contain from 20 to 60 constituents in various percentages, with two or three primary constituents (20–70%) [95].

Several studies have pointed out the role of EOs in tumor progression, the suppressive effect of EOs against a wide variety of cancers, and their use as adjunct therapy for multiple forms of cancers. Their effects are mostly accredited to the monoterpeneoid and sesquiterpeneoid compounds [96,97,98,99]. Aloe-emodin (1,8-dihydroxy-3-hydroxymethyl-9,10-anthracenedione), a hydroxyanthraquinone found in different Aloe species, has been reported to reduce cell proliferation and invasion, thus presenting anti-metastatic activity. Multiple studies support the antiproliferative capacity of this natural compound, with the induction of apoptosis being associated with the down-regulation of the Bcl-2 gene and increased caspase activity and induction of differentiation in B16 cells. Essential oils related to plants belonging to the Asteraceae and Apiaceae families, in particular, could represent a novel adjuvant in malignant melanoma treatment, due to the cytotoxic activity they presented against B16F10 cells [100]. Additionally, the anti-melanogenic effects of *Casearia sylvestris* Sw. EO and its main compound α-zingiberene against murine melanoma cell line B16F10 were demonstrated [52], suggesting that this sesquiterpene is most probably the one responsible for the cytotoxic activity.

In a seminal paper in 2017, it investigated the anti-cancer activity of *Plantago depressa* ethanolic (PDE) extract containing a high concentration of iridoid glycosides on B16F10 murine melanoma cells, stressing that the extract possesses anti-neoplastic properties, with treatment with a dose of 5 µg/mL leading to a major decrease in skin cancer cell viability (31.4; 34.0; 20.5% at 24, 48 and 72 h after incubation, respectively) [50].

Cannabidiol obtained from *Cannabis sativa* decreased metabolic activity of B16F10 cells and promoted cell death on the same cell line [51]. The chemo-preventive properties of cannabidiol are also supported by more recent studies [101] that emphasize its cytotoxic activity in B16F10 murine melanoma cells following the treatment with different concentrations (10, 20, 40, and 80 μg/mL). Another study [54] showed the cytotoxic effect of *Pachycereus marginatus* hexane, chloroform, methanol, and methanol-aqueous partition stem extracts, with an inhibition of viability up to 51% following treatment with chloroform extract at concentrations of 50 µg/mL.

## 5. Bioactive Natural Compounds Tested In Vivo Models

Animal models are widely employed in scientific study, particularly in biomedical research, to better our understanding of current health challenges and, more importantly, to advance the field. These models have aided in the comprehension of toxicological, pharmacokinetic, and surgical studies. The purpose of this section is to underline the importance of in vivo experiments with diverse phytochemicals.

### 5.1. Polyphenols

Gastaldello and collaborators (2021) studied the tumoral behavior of melanoma after therapy with two polyphenols, baccharin and p-coumaric acid, both isolated from Brazilian green propolis, in Balb/C mice implanted with B16F10, and showed an important decrease in tumor volume (236.4 ± 89.64), especially in the group treated with p-coumaric acid [10]. The number of mitotic events was also decreased in both of the groups that received the bioactive compounds, with the results being 1.356 ± 0.2475 for the p-coumaric group and 1.567 ± 0.2554 for the baccharin group, compared to melanoma control group (3.486 ± 0.2554). Moreover, the number of vessels obtained via optical microscopy was lower in the treated groups than in the control one, hence contributing to angiogenesis modulation.

### 5.2. Alkaloids

The Brazilian group of Garcia and collaborators published in 2020 a study [56] reporting that hydroalcoholic extract of the *Lobelia inflata* plant reduced the recruitment of inflammatory cells, edema, and inflammatory cytokines (IL-1, IL-6, and TNF-α), and also significantly reduced tumor growth after administration at 300 mg/kg. The alkaloids were reported by the researchers to be the main active substances in the obtained extract, and thus they can be considered to be responsible for the reported effects.

### 5.3. Terpenes

Mistletoe extract and the combination of mistletoe extract and triterpenoids have been reported [57] to possess anti-tumor effects. The main effects pointed out are the reduction of tumor surrounding blood vessels and necrosis induction. According to the researchers, following treatment with VCD (aqueous mistletoe extract with cyclodextrin), tumor growth was significantly stalled, and median survival was increased by 8.5 days, with one complete remission and two temporary tumor remissions. The VTT (triterpenoid aqueous mistletoe extract) group showed increased anti-neoplastic effects, with two complete remissions and six temporary remissions. In addition, an important decrease in CD31-positive tumor blood vessels was reported after treatment with solubilized triterpenoids and various mistletoe extracts.

### 5.4. Multiple Bioactive Compounds

*Bauhinia variegata* L. hydromethanolic extract has been reported to have a critical impact in forestalling B16F10 melanoma growth in tumor-bearing C57BL/6 mice [58]. Plant materials including leaves, stem bark, and floral buds were used to obtain the desired extract, and doses of 500 and 750 mg/kg were administered to the animals. The extracts were administered as adjuvants in cyclophosphamide therapy, resulting in a decrease in tumor volume, especially in the case of leaf extract (750 mg/kg) and flower extract (500 mg/kg). Additionally, the antioxidant levels were restored, and the GSH content was increased in tumor group animals after treatment with B. variegata, which could be related to the plant’s antioxidant and free radicle scavenging abilities. Also, protein-bound polysaccharide (PSK) was reported as an inhibitor of lung metastases of B16F10 melanoma induced to C57BL/6 mice [102].

## 6. Bioactive Compounds Tested Both In Vitro and In Vivo

In vivo testing is an important part of medical research in general, notably in clinical trials. In vivo investigations are useful for learning about the effects of a chemical or the progression of a disease in a whole, living creature. Furthermore, researchers use animal experiments to elucidate the underlying mechanisms of various disease processes and evaluate the safety of new therapies.

### 6.1. Polyphenols

Curcumin represents an intensively studied compound due to its anti-inflammatory, antioxidant, and anticancer properties. Multiple studies have been published on this matter, with results even suggesting that curcumin can significantly inhibit B16F10 cell proliferation by inducing a decrease in the cell number in the G1 phase. A group of scientists have highlighted the important effects of curcumin in the fight against B16f10 melanoma [59]. In vivo, they point to the anti-metastatic properties of curcumin as an important result. Other efficient polyphenols were described by Almeida and collaborators (2020), who reported in their study a list of plants that were proven to possess cell growth inhibition (GI) activity against B16F10 murine melanoma cells [60]. Among the plants specified, the most efficient extracts were obtained from the following: leaves of *Acnistus arborescens* (L.) Schltdl. (Solanaceae family), with a GI% of 91%, leaves of *Maclura tinctoria* (L.) D.Don ex Steud. (Moraceae family), with a GI of 82%, leaves of *Casearia sylvestris* Sw. (Salicaceae family), with a GI% of 85%, and leaves and stems of *Athenaea velutina* (Sendtn.) D’Arcy (Solanaceae family), with a GI of 84% in the case of leaves extract and 77% in the case of stems extract. The most potent cytotoxic extracts had their anti-metastatic activity determined through in vitro assays and using melanoma mouse models. Organic extracts of *Athenaea velutina* (EAv) leaves significantly suppressed B16F10 cell migration, adhesion, infiltration, and cell colonization. Five phenolic acid compounds were identified: three simple phenolics, quinic acid and its caffeic acid derivatives—caffeoylquinic acid and dicaffeoylquinic acid; and two flavonoids, kaempferol-3-*O*-rutinoside and quercetin-3-*O*-rutinoside. EAv noticeably suppressed the development of pulmonary melanomas following the intravenous injection of melanoma cells to C57BL/6 mice.

It was determined in 2015 that lipid-soluble ginseng extract (LSGE) has a significant ability to inhibit the invasion and migration of B16F10 melanoma cells in vitro [62]. Thus, following treatment with 3 µg/mL of LSGE, inhibition of invasion and migration by 98.1% and 71.4%, respectively, was reported. Moreover, lung metastasis of cancer cells was inhibited by 59.3% after treatment with 1000 mg/kg/day LSGE orally administered to C57BL/6 mice that had previously been injected in the tail vein with B16F10 cells. The authors concluded that LSGE possesses anti-invasive and anti-migratory activity by inhibiting MMP-2 expression, leading to a reduction in MMP-2 protein levels and activity. Cancer cell invasion and migration into adjacent tissues are mediated by MMP-2 and MMP-9. Consequently, regulation of MMP-2 and MMP-9 are crucial for preventing cancer invasion and metastasis. The antitumoral effect of LSGE was attributed to the main components, with polyphenolics and polyacetylenes, panaxynol (the dominant one), panaxydol, and panaxytriol being the main polyacetylenes responsible for this presumed effect.

### 6.2. Flavonoids

Flavonoids are very often correlated with chemopreventive, antiproliferative and pro-apoptotic activity, both in vitro and in vivo. Oroxylin A is reported to be one of the flavonoids that possesses the ability to suppress MMP-2 and -9 activity, hence abolishing B16F10 melanoma lung metastasis [103]. The impact of total oligomer flavonoids (TOF), containing chlorogenic acid, epicatechin, rutin, hyperoside, vitexine-2″-*O*-rhamnoside, procyanidin B2, procyanidin C1, oleanolic acid, and ursolic acid, isolated from the leaves of *Crataegus azarolus*, was assessed by Mustapha and collaborators in 2016 in vitro and in a Balb/c mouse model. Following B16F10 incubation for 48 h with a dose of 400 µg/mL, cell viability decreased by 77.57%. Moreover, these flavonoids exerted an antitumor effect, by significantly decreasing the tumor weight and volume in the TOF+melanoma group (0.24 g and 242 mm^3^) compared to the melanoma group (1.5 g and 3653.75 mm^3^). Subsequently [38], they demonstrated that the aqueous extract of *Daphne gnidium* leaves exhibited an important antiproliferative effect against B16F10 melanoma cells, while Chaabane and collaborators (2016) went ahead to assess the anti-neoplastic effect of the same extract in vivo, in a Balb/c mouse model [55]. According to the authors, inhibition of melanoma proliferation reached a maximum of 65.78% and 63.98% after 48 h of incubation with 70 μM of luteolin-7-glucoside and daphnetin, respectively. A remarkable result was reported after testing the extract in vivo, with the tumor volume being inhibited to a degree of up to 79.44%. Moreover, spontaneous proliferative capacity of the splenic lymphocytes was restored after treatment with aqueous extract in a dose of 200 mg/kg, with the stimulation percentage reaching 64.2%.

According to Tavakoli and co-workers (2018), curcumin (Cur), the active flavonoid ingredient of the turmeric (*Curcuma longa*) rhizome, and chrysin (Chr) possess important anti-proliferative and anti-metastatic effects on B16F10 murine melanoma in vitro and in vivo (C57B16 mice) [64]. Several studies have previously been conducted in this direction [104,105,106,107], demonstrating important properties like inhibition of tumor proliferation, invasion, metastasis, and angiogenesis, as well as antioxidant, anti-inflammatory and anti-apoptosis effects in numerous forms of neoplasia [108,109]. In addition, Tvakoli’s research demonstrated that these effects appear to be enhanced by encapsulating the natural compounds into PLGA-PEG NPs, also improving the safety of treatment delivery and enabling directed drug delivery to target tissues without damaging the healthy ones. Following the intraperitoneal injection of pure Cur (15 mg/kg), nano-encapsulated Cur (30 mg/kg), pure Chr (15 mg/kg), nano-encapsulated Chr (30 mg/kg), pure Cur and Chr (15 mg/kg, each of them) and nano-encapsulated Cur and Chr (30 mg/kg, each of them), respectively, on a daily basis for 12 days, a significant difference in tumor size was noticed compared to the control group. Hence, CurChr NPs displayed the best therapeutic performance. Chrysin was also reported [109] to be a potential antitumor agent after being tested in vitro and in vivo. Following in vitro treatment of B16F10, cell growth inhibition, apoptosis and cell cycle arrest at the G2/M phase were the effects observed. After receiving a dose of 50 mg/kg b.w. for 14 days and 21 days, when testing the BALB/c mice, a significant inhibition of tumor size was observed compared to the control group.

Experiments on B16F10 metastatic murine melanoma, testing *Citrus unschiu* peel ethanolic extracts (EECU), were conducted in 2019 [65], revealing an important cell growth inhibition and a markedly reduced cell viability, with apoptotic cells representing 26.65% and 48.19% at doses of 50 and 100 μg/mL, respectively. Moreover, following the assessment of the effect of EECU on metastatic activity, cell mobility was completely suppressed to 16.33% when treated with 40 μg/mL of EECU via regulation of MMP-2 and -9 expression, with naringenin and hesperidin being considered to be responsible for these effects.

Sanggenol L—a natural flavonoid—inhibited the growth of cells tested and induced significant morphological changes and alterations, revealing the cell growth inhibitory effect of sanggenol L. Additionally, this flavonoid has been demonstrated to induce apoptosis in B16F10 melanoma cells. Resveratrol exerted significant suppression on the growth of B16F10 melanoma, and also increased apoptosis. Moreover, numerous in vitro studies have indicated that resveratrol is able to induce autophagy, inhibiting the Akt/mTOR pathway in B16F10 melanoma cells. It has also been shown that resveratrol suppresses the growth of B16F10 murine melanoma cells and A375 human cells by promoting autophagy and inhibiting the PI3K/AKT/mTOR signaling pathway [70].

### 6.3. Alkaloids

Two alkaloids, coumarin and pyrrole 2-carbaldedhyde, extracted from *Angelicae dahuricae* Radix, presented anti-melanoma properties [66]. Migration, invasion, and colony formation of B16F10 cells were affected, with nuclear fragmentation and chromatin condensation being reported.

### 6.4. Terpenes

Jolkinolide B, a diterpenoid found in the root extract of *Euphorbia fischeriana Steud*, induces tumor cell apoptosis and decreased the level of ATP. Condensation of cytoplasmic and nuclei volume were the morphological changes after 24 h of incubation with the extract. A high percentage of apoptotic cells (50.11 ± 2.01%) compared to the untreated group (0.27 ± 1.13%) was reported. In vivo, in a C57BL/6 mouse bearing melanoma model, a significant tumor growth inhibition was reported: 17.3%, 34.6% and 54.4% in JB-treated groups with 10, 20 and 40 mg/kg [11]. The same authors [68] conducted another study, this time investigating *Juniperus communis* extract, which was described as a tumor growth suppressor and an apoptosis promotor on the basis of both in vivo and in vitro studies. Cell proliferation suppression, cell cycle arrest, angiogenesis and metastasis decrease, and weakening of cancer stem cell-associated marker expression were among the effects listed by the authors as a result of extract testing. The in vivo effects presented showed that a dose of 200 mg/kg, administered subcutaneously every 2 days for 7 days led to an average tumor size at day 23 of 861.84.13 ± 245.44 mm^3^ for the Juniperus extract-treated group, compared to 1573.89 ± 385.37 mm^3^ for the vehicle group. In addition, the survival rate was influenced, with the mice from the treatment group living significantly longer (35 days vs. 25 days for the control group).

Himachalol (7-HC), a major sesquiterpene isolated from *Cedrus libani*, possesses significant chemo-preventive properties. There is recent reported evidence [69] demonstrating the ability of this natural compound to inhibit B16F10 cell proliferation and to induce apoptosis by inducing cell cycle arrest, increasing the proportion of cells in the sub-G1 phase, and substantially dropping the S and G2/M phases. In addition, down-regulation of the anti-apoptotic protein Bcl-2 and an increase in the expression levels of the pro-apoptotic protein Bax have been reported.

In vivo, Ginsenoside Ro, an important saponin abundant in oleanolic acid, isolated from ginseng (*Panax ginseng*), inhibited tumor growth in B16F10 tumor-bearing C57BL/6J mice following treatment with a dose of 25 mg/kg Ro, although no effect on melanoma cells in vitro was noticed. On the other hand, Ro’s metabolites, Zingibroside R1, chikusetsusaponin IVa, and calenduloside E, showed anti-tumor effects both in vitro and in vivo [68].

### 6.5. Multiple Bioactive Compounds

In a recent review [70], the cytotoxic effect of anthocyanin and anthocyanidin extracts from blueberry fruits on B16F10 melanoma was emphasized, with anthocyanidins showing a stronger inhibitory effect. These results were also confirmed by in vivo testing. Another polyphenol, chrysin, suppressed melanoma growth by up to 71% and amplified the cytotoxic activity of NK cells, cytotoxic T lymphocytes, and macrophages. The same authors reported elderberry to contain an important concentration of anthocyanins that showed anti-proliferative effects in a melanoma model. In this study, LDH level was influenced following the administration of a dose of 250 µg/mL anthocyanin-enriched extract, inducing an increase in LDH by 74% compared to the control group. A team of researchers from Mexico [71] reported *Cuphea aequipetala* to be a plant that possesses important compounds with the ability to induce cytotoxicity through the apoptosis pathway in B16F10 cells. Moreover, following the oral administration of aqueous extract and methanolic extract, respectively, from the same plant to C57BL/6 female mice, for 14 days after melanoma induction, a significant reduction in tumor size was observed (up to 80% with the aqueous extract, and 31% with the methanolic extract). In 2018, powerful evidence was provided for the first-time [72] supporting the ability of GBEE (*Ginkgo biloba* exocarp extract), with doses ranging from 50 to 200 mg/kg used in their in vivo study, to inhibit the growth of B16F10 melanoma transplanted tumor, and in addition that it possessed an anti-pulmonary metastatic effect. This in vivo research was supported by a previous in vitro investigation that showed treatment with doses between 5–320 µg/mL to have a significant inhibitory effect in a concentration-dependent manner on cell proliferation as well as on the migration and the heterogeneous adhesion of B16-F10 to HUVEC. GBEE (50–200 mg/kg) showed a powerful reducing capacity of MMP-9 expression, a protein that has increased expression and activity in metastatic tumors. Depending on the dosage, in vitro, this substance suppresses the development of tumor cell invasion and their adherence to constituents of the basement membrane [110,111].

*Pyrostegia venusta* flower heptane extract (HE) has been reported to induce apoptosis in melanoma cells. The mechanisms through which apoptosis is induced seem to be the disruption of the mitochondrial membrane potential and induction of ROS. Membrane blebbing, cell shrinkage, chromatin condensation and DNA fragmentation and activation of caspase-2, -3, -8, -9 have also been reported to be related to treatment with *Pyrostegia venusta* extract. HE treatment produced an increase in cell cycle arrest at G2/M phases in the murine melanoma cells. The main fractions of HE separated by the researchers, octacosane and triacontane, were correlated with cytotoxic activity against B16F10 melanoma cells in vitro. Moreover, the same fractions proved to be potent protective agents against subcutaneous melanoma in vivo [73].

### 6.6. Carotenoids

Carotenoids are natural pigments found in plants and animals, their number exceeding 700. They have been credited with many beneficial effects, including antioxidant activity as well as the prevention and slowing of the development of certain diseases (degenerative disease, cardiovascular diseases and various types of cancer).

An important compound, found this time in the brown algae fucoxanthin, has been reported by many experts as a promising agent in the fight against malignant melanoma [74,75]. This carotenoid was effective in vitro and also in vivo in Balb/c mouse model. In vitro, fucoxanthin showed an increased ability to inhibit cell proliferation by up to 87%, presenting apoptotic bodies and nuclear condensation. Cell cycle arrest and apoptosis were observed following the treatment of B16F10 cells, with activation of caspase-3 and -9 and down-regulation of Bcl-Xl expression being considered the mechanism of action, while in mice, tumor growth was strongly inhibited, with a 5-fold reduction following fucoxanthin administration. Moreover, inhibition the formation of lung metastasis has also been reported, and is considered an important effect [112].

### 6.7. Peptides

Plant defensins are cationic peptides found throughout the plant kingdom that belong to a large superfamily of antimicrobial peptides found in a variety of organisms collectively known as defensins.

Psd1 is a pea plant defensin belonging to this superfamily of cationic peptides. Amaral and collaborators (2020) investigated the effect of Psd1 on tumor cell viability, and the results showed a significant inhibition of viability in a dose-dependent manner after a treatment of 24 h, with a 50 to 60% reduction in the cells when treated with 25 and 50 µM of the peptide [76]. The mechanism by which psd1 induced apoptosis was by altering the integrity of the cellular membrane in a time-dependent manner, most probably accompanied by processes as oxidative stress of mitochondria. One of the hallmarks of apoptosis, internucleosome DNA fragmentation, was also exhibited in a high proportion, supporting the hypothesis that the decrease in cell viability of murine melanoma cells is associated with apoptosis. In addition, psd1 has been reported to have a strong effect on B16F10 tumor metastasis in vivo, in a mouse model, after treatment with a dose of 3 mg/kg, completely abolishing tumor development, whereas a significant decrease in the number of pulmonary nodules, by 75% and 88%, was noticed in the animal groups treated with 0.5 mg/kg and 1 mg/kg Psd1, respectively.

## 7. Conclusions

Mother Nature is an essential source of bioactive natural products. The efficient diversity of phytochemicals is ready to supply unique and renewable resources to find and develop potential new anticancer agents, indicating that the investigation of the biological and pharmaceutical properties of medicinal plants and their natural products is a crucial issue in the development of complementary therapies.

Diverse mechanisms and pathways are controlled by the anti-melanoma activity of natural bioactive nutrients, like increased apoptosis, caspase activity, and expression of p53- and Bcl-2-associated X (bax), and also the inhibition of angiogenesis and tumor-promoting proteins, showing the complexity and importance of natural compounds in the chemoprevention and therapy of murine malignant melanoma. These findings are an important step forward in medical research, as they can help science evolve to identify new methods of chemoprevention and therapies for melanoma, in both animals and humans.

## Figures and Tables

**Table 1:** Bioactive compounds tested against B16F10 murine melanoma.

No.	Plant Name	Common Name	Part Used	Type of Extract	Most Important Bioactive Components	Dose Concentration	Mechanism	Reference
Bioactive Compounds Tested In Vitro								
***Polyphenols***								
**1.**	*Equisetum ramosissimum*	Branched scouringrush	Whole plant	Ethyl acetate, dichloromethane, n-hexane, methanol, and water extracts	Polyphenols	5, 50, 100, and 200 μg/mL	activation of caspase-3 and -9; tyrosinase suppression; MITF, Trp-1, and Trp-2 regulation	[22]
**2.**	*Cynomorium coccineum* L.	Maltese Mushroom	Whole plant	Aqueous extract	cyanidin 3-*O*-glucoside; gallic acid	25 to 500 μg/mL	anti-tyrosinase activity;	[23]
**3.**	*Cucurbita maxima* Duch	Autumn squash, marrow, pumpkin, turban gourd, buttercup squash	Hull-less pumpkin (HLP) and hull pumpkin (HP)	Ethanolic extracts	Coumaric acid	HLP polyphenols extract (10, 20, 40, 60, 80, 100, 200, 400, 600, 800, or 1000 μg/mL)	↓ tyrosinase; ↓ intracellular melanin	[24]
**4.**	*Moringa oleifera* Lam., *Eremomastax speciosa* (Hochst.) Cufod and *Aframomum melegueta* K. Schum	Horseradish tree; Yoruba; Grains of Paradise	Leaves and seeds	Aqueous extracts	Not mentioned	2 mg/mL	↑ G2/M phase; arrest of the cell cycle in G1 phase; ↑ p53; ↑ p^21WAF1/Cip1^;	[25]
**5.**	*Syringa vulgaris* L.	Common lilac	Flowers, leaves, bark and fruit	Ethanolic extracts	Acteoside and echinacoside; ligstroside, syringalactone A, oleuropein-aglycone.	leaves (11.34–56.7 µmol GAE/mL), fruit (6.66–33.2 µmol GAE/mL), bark (9.875–49.37 µmol GAE/mL) and flowers (11.69–58.475 µmol GAE/mL)	Antioxidant and cytotoxic activity	[26]
**6.**	*Caesalpinia spinosa*	Spiny Holdback, Tara	Whole plant	Ethanolic extract	P2Et	72.1 μg/mL	↑ cell death; inducing of autophagy	[27]
**7.**	*Anastatica hierchuntica*	Rose of Jericho	Root and leaves	Methanolic extract	kaempferol, luteolin, quercetin	200–1000 μg/mL	↓ mitochondrial membrane potential; ↓ GSH; ↓ ROS;	[28]
**8.**	*Abeliophyllum distichum Nakai*	White forsythia	Leaves	Methanolic extract	acteoside, eutigoside B, isoacteoside, rutin, cornoside, hirsutrin, chlorogenic acid, caffeic acid, gentisic acid, ferulic acid, and quercetin	50–200 µg/mL	↑ ROS in cancer cells; ↑ caspase −3 and −9; ↓ Bax/Bcl-2 ratio; activation of MEK 1/2 and ERK 1/2	[29]
**9.**	*Phoenix dactylifera* L.	Date palm	Seeds	Aqueous extract	Ferulic acid	0.245 and 0.49 (mg/mL)	↓ ROS; ↓ melanogenesis signal proteins: p-p38, p-JNK, p-ERK, and p-CREB;	[30]
**10.**	*Origanum vulgare* L.	Oregano	Whole plant	Hydroalcoholic extract	Chrysin, quercetin-3-*O*-arabinoside, rutin	10 mg/mL	mitochondria and DNA damage; ↑ the number of cells in G2/M phase; ↓ expression of CCNB1 and CDK1 genes;	[31]
**11.**	*Remirea maritima*	Beachstar	Whole plant	Hydroalcoholic extract	Vitexin, isovitexin and luteolin	0.1–100 μg/mL	hydroxyl radicals scavenging;	[32]
**12.**	*Spartium junceum* L.	Spanish broom, weaver’s broom	Flowers	Aquaeous extracts and Hydroalcoholic extract	Multiple polyphenols	2, 4, 6, 8, 10 HFE mg/mL	↓ cell proliferation	[33]
**13.**	*Viscum album*	Mistletoe	Whole plant	Ethanolic extract	caffeic acid, chlorogenic acid, sakuranetin, isosakuranetin, syringenin 4-*O*-glucoside, syringenin 4-*O*-apiosylglucoside, alangilignoside C and ligalbumoside A	1 to 5% *v*/*v*	DNA fragmentation; ↑ Sub G0 population; ↑ S and G2/M populations;	[34]
***Flavonoids***								
**14.**	*Perilla frutescens var. crispa*	Purple shiso, Chinese basil and purple perilla.	Leaves	Ethanolic extract	Apigenin and sinensetin	25 µg/mL Pfc 5	↓ tyrosinase activity; ↓ LPS-induced pro-inflammatory genes	[35]
**15.**	*Punica granatum*	Pomegranate	Peel	Ethanolic extract	Kaempferol, luteolin, quercetin and proanthocyanidin, complex polysaccharides. hydrolyzable tannins (ellagitannin, punicalagin, punicalin and pedunculagin).	10–450 µg/mL	↓ cell proliferation and angiogenesis; ↓ VEGF gene;	[36]
**16.**	*Lindera obtusiloba*	Blunt-Lobed Spicebush	Leaves	Methanolic extract	Quercitrin and afzelin	100–1000 µg/mL	↓ tyrosinase activity; ↓ melanin synthesis; ↓ MITF and tyrosinase proteins; activation of MAP kinase pathway	[37]
**17.**	*Daphne gnidium*	Flax-leaved daphne	Leaves	Aqueous extract	Daphnetin and luteolin-7-glucoside	70 μM of luteolin-7- glucoside and daphnetin	activation of caspase 3; inducing sub-G1 cell cycle arrest;	[38]
***Anthocyanins***								
**18.**	*Sambucus nigra*	Elderberry	Fruits	Aqueous extract	Cy-3-*O*-samb; Cy-3-*O*-samb-5-gluc; Cy-3,5-digluc; Cy-hexoside-pentoside; Cy-3-*O*-gluc	250 µg/mL	↑ LDH; detachment, rounding up, shrinkage, and blebbing of membrane and apoptotic bodies;	[39]
**19.**	*Vaccinium uliginosum* L.	Low-bush blueberryes	Fruits	Hydroalcoholic extract	Anthocyanins and anthocyanidins	12.5–800 μg/mL (IC50 = 134.1 and 206.7 μg/mL)	Blockage of cell cycle procession at the G0/G1 phase; down-regulation of cyclin D1 expression; ↓ caspase-3 and p53;	[40]
**20.**	*Vaccinium* spp.	Blueberry	Fruits	Methanolic extract	Delphinidin glycosides, cyanidin glycosides, petunidin glycosides	200–750 μg/mL	↑ LDH, cell detachment, rounding up and shrinkage,	[41]
**21.**	*Vaccinum* spp., *Ribes nigrum*	Blueberry and blackcurrants	Fruit juice	Methanolic extract	delphinidin-3-*O*-glucoside, delphinidin-3-*O*-rutinoside, cyanidin-3-*O*-glucoside, cyanidin-3-*O*-rutinoside	0–500 μg/mL (blueberry); 0–350 μg/mL (blackcurrants)	Antiproliferative effect	[42]
***Alkaloids***								
**22.**	*Piper nigrum*, *Piper longum*	Black and long pepper	Fruits	Not Mentioned	Piperine	Not Mentioned	G1 Phase Cell Cycle Arrest; ↑ ROS in tumor cells	[43]
***Polysaccharides***								
**23.**	Malus domestica Borkh	Apple pomace	Dried apple pomace	Enzymatic preparations	Pectin	1 mg/mL	↓ adhesion, proliferation and cell invasion;	[44]
**24.**	*Inonotus obliquus*	Chaga	Fruits	Aqueous extracts	Polysaccharide	1–1000 μg/mL	↓ MMP-2 and MMP-9; ↓ NF-κB signaling pathway; ↓ migration ability	[45]
***Terpenes***								
**25.**	*Callicarpa longissimi*	Beautyberry	Leaves	Ethanolic extract	Carnosol and carnosic acid	10 μg/mL	↓ MITF gene expression	[46]
**26.**	*Gelidium latifolium*	Red Macroalgae, Tengusa, makusa, genso.	Whole plant	Ethanolic extract	Brassicolene	100–200 µg/mL	↓ Bcl2; ↑ p53, Bax, and Bak expression	[47]
**27.**	*Olea europeae* L.	Common olive	Olive pomace	Not mentioned	Maslinic acid	0 to 212 µM	↓ ROS;	[48]
**28.**	*Holothuria leucospilata*	Black sea cucumber, black tarzan	Body wall	Ethanolic extract	Saponin	(0, 4, 8, 12, 16, 20 μg/mL) and dacarbazine (0, 1200, 1400, 1600, 1800, 2000 μg/mL)	↑cells in sub-G1 peak; activation of caspse-3 and caspase-9;	[49]
**29.**	*Plantago depressa Wild*	Plantains or fleaworts	Whole plant	Ethanolic extract	Aucubin and iridoid glycoside	0.6, 1.2, 2.5 and 5 µg/mL	Apoptosis with cell morphology changes	[50]
**30.**	*Cannabis sativa*	Hemp, grass, hashish,	Flowers and leaves	CO_2_ and standardized based on 4% cannabidiol	Cannabidiol	25, 12.5, and 6.25 µg/mL	↓cell viability	[51]
***Essential Oils***								
**31.**	*Casearia sylvestris*	Wild sage	Leaves	Essential oil	α-zingiberene	10 mg/mL	cytotoxic activity	[52]
***Multiple classes of compounds***								
**32.**	*Zanthoxylum rhetsa*	Indian prickly ash	Bark	Methanolic extract	Yangambin and kobusin, columbamine and lupeol	500, 250, 125, 62.5, 31.25, 15.625, 7.81 µg/mL	Cytotoxicity	[11]
**33.**	*Crataegus azarolus*	Azarole, azerole, and Mediterranean medlar.	Leaves	Ethyl acetate extract	Ursolic acid and vitexin-2′’-*O*-rhamnoside	400 µg/mL	↓ cell proliferation	[53]
**34.**	*Pachycereus marginatus* (DC.) Britton & Rose	Organ-pipe cactus	Stem	Hexane, chloroform, methanol, and aqueous methanol extracts	Not mentioned	0.03 to 500 µg/mL	Cytotoxicity	[54]
**Bioactive Compounds Tested in In Vivo Models**								
***Polyphenols***								
**35.**	*Baccharis dracunculifolia*	Green propolis	Whole plant	Hydroalcoholic extract	Baccharin and p-coumaric acid	500 μg/kg	↓ cell mitosis; ↓ angiogenesis;	[9]
***Flavonoids***								
**36.**	*Daphne gnidium*	Flax-leaved daphne	Leaves	Aqueous extract	Daphnetin and luteolin-7-glucoside	200 mg/kg for 21 days	↓ tumor growth; restoration of the proliferation of splenic lymphocytes;	[55]
***Alkaloids***								
**37.**	*Lobelia inflata*	Indian tobacco	Bark	Ethanolic extracts	Alkaloids	300 mg/kg, orally administered, for 30 days	↓ tumor growth; ↓ IL-1, IL-6 and TNF-α	[56]
***Terpenes***								
**38.**	*Viscum album* L.	Misteltoe	Whole plant	Aqueous extract	oleanolic acid	12 µg/kg ML-I	↓ angiogenesis; ↑ caspase-3;	[57]
***Multiple Compounds***								
**39.**	*Bauhinia variegata* linn.	Orchid tree	leaves, stem bark and floral buds	Hydro-methanolic extract	alkaloids, flavonoids, tannins, terpenoids and glycosides	500 and 750 mg/kg	↓ tumor volume; ↑ GSH;	[58]
**Bioactive Compounds Tested Both In Vitro and in In Vivo Models**								
***Polyphenols***								
**40.**	*Curcuma longa* L.	Turmeric	Rhizomes	Not mentioned	Curcumin	1.10–270 µM	Modulation of BCl2, MAPKS, p^21^ and some microRNAs; NF-jB, IKKmodulation	[59]
**41.**	*Athenaea velutina*	Athena Sendtn.	Leaves	Organic solvent mixture (dichloromethane/methanol, 1:1) and distilled water	quinic acid, and its caffeic acid derivatives—caffeoylquinic acid and dicaffeoylquinic acid; kaempferol-3-*O*-rutinoside and quercetin-3-*O*-rutinoside.	1.562–200 μg/mL;	↓ migration, adhesion, invasion and cell colony formation	[60]
						100 mg/kg bw, daily, for 21 days		
**42.**	*Lithospermum erythrorhizon*	Purple gromwell; red gromwell	Roots	Hexane extract	Shikonin; Deoxyshikonin; b-Hydroxyisovalerylshikonin; Acetylshikonin and Isobutyrylshikonin	0, 0.5, 1, 2, 3 and 4 µg/mL	activation of caspase 3; inducing sub-G1 cell cycle arrest; ↓ Bcl-2	[61]
						10 mg/kg/day, 21 days		
**43.**	*Panax ginseng*	Gingseng	Whole plant	Ethanolic extract	Polyacetylenes and polyphenolics	0.3, 1.0, 3.0, or 10.0 µg/mL	Activation of caspase-8 and -9; inhibition of transcription of MMP-2 DNA;	[62]
						Orally administration of 300 and 1000 mg/kg in 0.2 mL of 4% ethanol solution once per day for 13 days		
***Flavonoids***								
**44.**	*Crataegus azarolus L*	Hawthorn, Zâarour	Leaves	Aqueous extract	(−)-epicatechin (EC)	400 µg/mL (in vitro)	↓ intracellular melanin; ↓ DCFH production; ↓ tyrosinase activity	[63]
**45.**	*Curcuma longa*	Turmeric	Rhizome	Aqueous extract encapsulated	Curcumin and chrysin	pure Cur-Chr mixture (CurChr) (each of them 5, 10, 15, 20, 30, 40, 50 and 60 lM) and equivalent concentrations of encapsulated Chr and Cur mixture (CurChr NPs)	↓ MMP-9, MMP-2 and TERT genes expressions; ↑ TIMP-1 and TIMP-2; ↓ tumor growth;	[64]
						pure Cur (15 mg/kg), nano-encapsulated Cur (30 mg/kg), pure Chr (15 mg/kg) and nano-encapsulated Chr (30 mg/kg)		
**46.**	*Citrus unshiu*	Miyagawa mandarin, unshu mikan	Peel	Ethanolic extracts	Naringin and hesperidin	0, 20, 40, 60, 80, and 100 μg/mL	↓ mitochondrial membrane potential; ↑ LDH; ↑ ROS; ↓ migration, invasion, and colony formation	[65]
						100 μL of 100 mg/kg/day; 100 μL of 200 mg/kg/day for 21 days	↓ LDH, ↓ lung hypertrophy, the number and expression of metastatic tumor nodules	
***Alkaloids***								
**47.**	*Angelica dahurica Radix*	Chinese angelica, the garden angelica, root of the Holy Ghost	Root	Ethanolic extract	Coumarine and pyrrole 2-carbaldedhyde	100 μL of EEAD	↑ Bax/Bcl-2 expression ↓ MMP-2 and -9 expression	[66]
						100 mg/kg/day; 100 μL of EEAD 200 mg/kg/day	↓ LDH	
***Terpenes***								
**48.**	*Euphorbia fischeriana Steud*	Lang-Du	Root	Not mentioned	Jolkinolide B	20, 40 and 60 μM	↑ mRNA level of Bax ↓ mRNA levels of Bcl-2, Caspase-3 and Caspase-9 ↑ ROS	[10]
						10, 20 and 40 mg/Kg	↓ mRNA levels of Bcl-2 and Caspase9	
**49.**	*Panax ginseng* C.A. Meyer	Japanese ginseng	Not Mentioned	Not Mentioned	Ginsenoside Ro- Zingibroside R1, chikusetsusaponin IVa, and calenduloside E (Ro metabolites)	0, 1, 3, 10, 30, and 100 µg/mL	Without cytotoxic effect in vitro	[67]
						25 mg/kg i.p., 15 days	↓ tumor weight; anti-angiogenic activity	
**50.**	*Juniperus communis*	Common juniper	Not Mentioned	Distillation	α-Pinene, citronellyl acetate, and d-limonene	0–100 μg/mL	anoikis, chromatin condensation, DNA fragmentation, and the appearance of apoptotic bodies; ↓ bcl-2 and procaspase-9; ↑ bax; ↑ Fas and FasL expression; ↓ procaspase-3 and -8;	[68]
						200 mg/kg, sc, every 2 days, for 14 days	↓ tumor growth; ↑ survival rate	
**51.**	*Cedrus libani*	Cedar of Lebanon	Wood	Hexane extracts	Himachalol	1, 5, 10, 15 and 25 μg/mL	↑ sub-G1 phase ↓ S and G2/M phases ↓ Bcl-2, ↑ Bax; inhibition of MAPK/ERK and PI3K/AKT pathways;	[69]
						0.2 mL- topical administration, 0.02 mL of 7-HC, 50 mg/kg, diluted in sunflower oil—gavage administration, 0.1 mL dissolved in DMSO (10, 25, or 50 mg/kg)- ip administration	↓ tumor volume	
***Multiple Compounds***								
**52.**	Various plants	Various names	Various Parts of the Plants	Various types of Extract	Cyanidin (Cyanidin-3-*O*-glucoside), Anthocyanins enrich extract (delphinidin, cyanidin, petunidin, peonidin and malvidin), Hesperetin Apigenin, Baicalein and Baicalin, Diosmin Caffeic acid phenethyl ester (CAPE), Gallic acid, Resveratrol	Various doses	↓ tumor growth; ↓ cell proliferation; ↓ viability; ↑ apoptosis; ↑ oxidative damage; ↓ mitochondrial membrane potential	[70]
**53.**	Various Plants	Various names	Various Parts of the Plants	Various Types of Extract	Multiple Compounds	Various Doses	↓ MMP-2 and MMP-P expressions	[8]
**54.**	*Cuphea aequipetala*	Mexican Loosestrife	Aerial parts	methanolic and aqueous extracts	phenols, terpenes, steroids, and saponins	aqueous (0.05, 0.1, 0.2, 0.4, 0.6, 0.8 mg/mL) and methanolic extracts (0.0425, 0.085, 0.17, 0.34, 0.51, 0.68 mg/mL)	↑ cells in G1 phase; cytoplasm shrinkage; DNA fragmentation;	[71]
						25 mg/mL, in water, for 14 days	↓ tumor volume	
**55.**	370 plants	Various Names	Various Parts of the Plants	Various Types of Extract	Multiple Compounds	Various Doses	↓ cell proliferation	[4]
**56.**	*Ginkgo biloba*	Maidenhair tree	Exocarp	Ethanolic extract	Proteoglycan	5–320 µg/mL	regulation of PI3K/Akt/NF-kB/MMP-9 signaling pathway	[72]
						50–200 mg/kg		
**57.**	*Pyrostegia venusta*	Flamevine or orange trumpetvine	Flowers	Heptane extract	Octacosane and triacontane	Not Mentioned	disruption of the mitochondrial membrane potential and ↑ ROS; activation of caspase-2, -3, -8, -9; cell cycle arrest at G2/M	[73]
***Carotenoids***								
**58.**	*Ishige okamurae*	Brown algae	Whole plant	Not Mentioned	Fucoxanthin	Not Mentioned	Modulation of Bcl-2 proteins, MAPK, NFκB, Caspases, GADD45; cell cycle arrest in the G0/G1; DNA fragmentation; ↓ MMP-9; ↓ tumor growth;	[74]
**59.**	*Ishige okamurae*	Brown algae	Whole plant	Not Mentioned	Fucoxanthin	50, 100, and 200 µM	induction of cell cycle arrest during the G0/G1 phase and apoptosis; ↓ Rb; ↓ cyclin D (1 and 2); ↓ CDK; ↑ p^15^INK4B and p^27^Kip; ↓ Bcl-xL; ↑ caspase-9, caspase-3, and PARP	[75]
						300 µg/100 µL/mouse adm. ip, every 5 days		
***Peptides***								
**60.**	*Pichia pastoris*	Yeast	Seeds	Water extract	Pisum sativum defensin	0, 3.12, 6.25, 12.5, 25, or 50 µM for 24, 48, or 72 h	↑ sub-G0/G1; DNA fragmentation; ↓ inflammatory cells;	[76]
						0.1–3 mg/kg		

## Data Availability

The data presented in this study are available on request from the corresponding author.

## Sample Availability

Samples of the compounds are not available from the authors.
